# Double Trouble: Exploring Bilateral Acetabular Fractures in a Trauma Patient

**DOI:** 10.7759/cureus.56889

**Published:** 2024-03-25

**Authors:** Elliott Smith, Anna C Deal, Saptarshi Biswas

**Affiliations:** 1 Surgery, Grand Strand Medical Center, Myrtle Beach, USA

**Keywords:** trauma patient, trauma and orthopedics, bilateral acetabular fracture, acetabular fracture, pelvic and acetabular fracture

## Abstract

Bilateral acetabular fractures, though rare, pose significant challenges in both diagnosis and management due to their association with high-energy trauma and the potential for long-term disability. This case report presents the clinical course of a 27-year-old female who presented to our department after a motorcycle accident with bilateral acetabular fractures. Initial assessment revealed nondisplaced bilateral acetabular fractures, along with associated injuries including a right ulnar styloid fracture. Further evaluation via 3D CT scan delineated associated column fractures on the right and posterior + anterior wall fractures on the left, classified according to the Letournel and Judet system. Notably, this specific combination of acetabular fractures has not been documented in existing literature as per our investigation. The surgical intervention involved an anterior intrapelvic approach for open reduction and internal fixation (ORIF) of the right acetabulum, while the left acetabulum was managed conservatively. Postoperatively, the patient is scheduled for non-weightbearing activity until radiographic evidence of fracture healing is observed. This case underscores the importance of tailored surgical approaches and comprehensive management strategies in optimizing outcomes for patients with bilateral acetabular fractures.

## Introduction

Bilateral acetabular fractures represent a rare and formidable subset of pelvic injuries, typically stemming from high-energy trauma incidents, and often accompanied by significant morbidity [1,2]. The complex anatomical structure of the acetabulum, which serves as the primary weight-bearing surface for the hip joint, comprises an anterior column and a posterior column. These include the anterior ilium, anterior wall and dome, superior pubic ramus for the anterior column and the posterior wall and dome, ischial tuberosity, and greater and lesser sciatic notches for the posterior column. This complexity underscores the critical need for prompt and precise management to optimize functional outcomes. Despite their infrequent occurrence, bilateral acetabular fractures demand meticulous attention due to their potential for long-term disability and complications [3].

While unilateral acetabular fractures have garnered considerable attention in the literature, bilateral fractures present additional challenges, necessitating a tailored approach to maximize patient outcomes [3]. The scarcity of comprehensive case reports on bilateral acetabular fractures highlights the importance of sharing individual experiences to enrich the collective understanding within the medical community.

We explore the unique anatomical and biomechanical considerations of bilateral acetabular fractures, including the associated risks and complexities in diagnosis and management. Additionally, we review existing literature to contextualize our findings and contribute to the collective understanding of this challenging orthopedic condition.

In this context, we present the case of a 27-year-old female who sustained bilateral acetabular fractures as a result of a motorcycle accident. Through an examination of the patient's presentation, diagnostic evaluation, surgical management, and postoperative outcomes, we aim to provide insights into the management of this rare and intricate injury pattern.

## Case presentation

A 27-year-old female was the passenger in a motorcycle accident and presented to the emergency department as an activated level 1 trauma with a blood alcohol level of 128. On the primary survey, the patient was awake and talking, indicating a patent airway. The patient had a symmetrical chest rise with an O2 saturation of 97% on room air. Chest x-ray in trauma bay showed no acute abnormalities and pelvic x-ray was positive for bilateral pelvic fractures. The patient was hemodynamically stable with a BP of 121/68 and a pulse of 82. E-FAST exam was negative for free fluid in the abdomen and pelvis, pneumothorax, and pericardial effusion. The patient had a Glasgow coma score of 14. 

**Figure 1:**
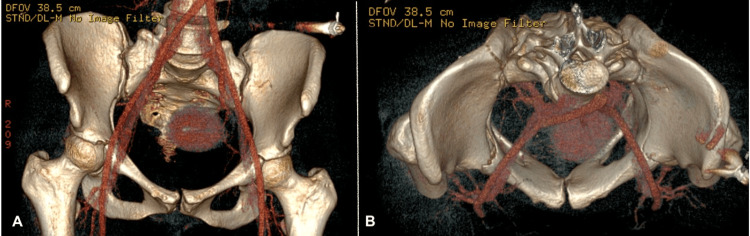
3D CT scan of bilateral acetabular fractures. (A) Anterior view of 3D CT scan showing right nondisplaced anterior, middle, and posterior acetabulum fractures and left nondisplaced anterior and middle acetabulum fractures. (B) Axial view of the pelvis from 3D CT scan.

On the secondary survey, road rash was present on the patient’s right lower extremity. The patient’s right lower extremity had positive log roll, pain with gentle rolling of the thigh internally or externally, and pain with hip flexion and extension. There was swelling and abrasions to the extremity, but sensation and motor strength were intact. The patient’s left lower extremity had negative log roll but discomfort on hip flexion and extension. The left lower extremity showed no swelling and sensation and motor strength were intact. The pelvis was tender to palpation, stable to compression, and had no gross instability. The patient also had swelling in the right wrist with abrasions over the digits. Right radial pulses were present along with sensation and motor strength. 3D CT scan of the pelvis showed right nondisplaced anterior, middle, and posterior acetabulum fractures and left nondisplaced anterior and middle acetabulum fractures (Figures 1A, 1B). After evaluation, the patient was counseled and consented to surgical stabilization of the right acetabulum and closed treatment of the left acetabulum. X-ray of the right upper extremity showed a right ulnar styloid fracture with displacement.

**Figure 2:**
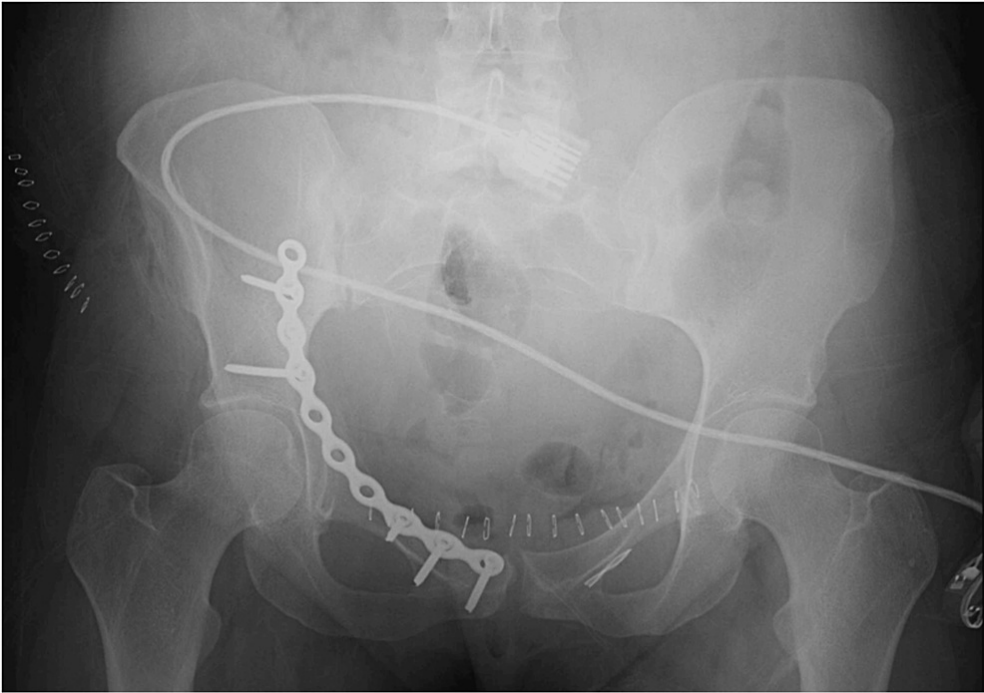
Postoperative x-ray of the pelvis showing placement of pelvic recon plate and screws.

The patient underwent open reduction and internal fixation (ORIF) on the right acetabulum with a pelvic recon plate placed (Figure 2).

The patient tolerated the surgery well. Postoperatively, the patient was non-weightbearing for eight weeks or until radiographic evidence of healing of the fractures. The patient continues to do well in clinic follow-up.

## Discussion

Bilateral acetabular fractures, particularly resulting from high-energy mechanisms such as motorcycle accidents, present a formidable challenge to orthopedic surgeons. The unique anatomical and biomechanical considerations of the acetabulum contribute to the complexity of these injuries, necessitating a comprehensive approach to diagnosis, classification, and treatment. In the case of our 27-year-old female patient who sustained bilateral acetabular fractures in a motorcycle accident, we explore the nuances of managing such injuries and discuss our findings considering existing literature.

The Letournel and Judet classification system remains the most used classification system for acetabular fractures [4]. In previous studies that analyzed the most common types of acetabular fractures based on the Letournel and Judet classification system, high-velocity acetabular fracture groups commonly presented with posterior wall and transverse posterior wall fractures. Bilateral acetabular fractures from high-velocity accidents most commonly presented with bilateral transverse posterior wall fractures or posterior wall and anterior wall fractures in 18% and 14% of cases respectively [1]. Our patient presented with associated column fractures on the right and posterior + anterior wall fractures on the left. This specific injury has not been reported in our research.

Past studies show most acetabular fractures have associated injuries due to the high-velocity nature of the fractures. The most commonly associated injury is in the lower extremity, followed up upper extremity (as seen in our patient), and pelvis/spine [1,5]. Acetabular fractures secondary to high-velocity injury are also associated with hip dislocation requiring emergency reduction [6]. The complexity of bilateral acetabular fractures lies in their presentation and management, as the bowel or urogenital system may be affected increasing the risk of contamination and infection [7].

A recent review of bilateral acetabular fracture cases showed that 45% of cases underwent ORIF, 40% were treated conservatively, and 15% underwent total hip arthroplasty (THA) [1]. ORIF remains the “gold standard” treatment for both unilateral and bilateral acetabular fractures. Conservative treatment is usually reserved for minimally displaced fractures, elderly with osteoporosis, or patients with comorbidities. However, THA is more commonly used for acetabular fractures from low-energy mechanisms, such as seizure patients, that begin as conservative treatment and undergo THA at a later time, or elderly patients with simultaneous femoral neck fractures [1,8]. Our patient underwent ORIF for the associated column fracture of the right acetabulum and closed treatment with manipulation for the left posterior and anterior wall fractures. A Modified Stoppa/anterior intrapelvic approach was used on our patient to allow for the best access to the right acetabulum. In incidences of high-velocity acetabular fractures, the Kocker-Langenbeck approach was most commonly used at 57% while Modified Stoppa followed at 18% [1].

The patient tolerated the surgery well and will be non-weightbearing bilaterally on her lower extremities for eight weeks or until radiographic evidence of healing fractures is present. To date, no post-operative complications have been reported, but the patient is still under follow-up.

## Conclusions

In conclusion, our case of a 27-year-old female patient with bilateral acetabular fractures from a motorcycle accident highlights the complexities of managing such injuries. Our tailored approach, including anterior intrapelvic ORIF for the right acetabulum and conservative management for the left, exemplifies the importance of personalized strategies in optimizing outcomes. Despite challenges like associated injuries and infection risk, our patient's successful postoperative course underscores the potential for favorable outcomes with comprehensive management. Continued research and sharing of clinical experiences are crucial for refining treatment approaches and improving patient care in similar scenarios, advancing orthopedic trauma management.
